# Staging Parkinson's Disease Combining Motor and Nonmotor Symptoms Correlates with Disability and Quality of Life

**DOI:** 10.1155/2021/8871549

**Published:** 2021-05-13

**Authors:** D. Santos García, T. De Deus Fonticoba, J. M. Paz González, C. Cores Bartolomé, L. Valdés Aymerich, J. G. Muñoz Enríquez, E. Suárez, S. Jesús, M. Aguilar, P. Pastor, L. L. Planellas, M. Cosgaya, J. García Caldentey, N. Caballol, I. Legarda, J. Hernández Vara, I. Cabo, L. López Manzanares, I. González Aramburu, M. A. Ávila Rivera, M. J. Catalán, V. Nogueira, V. Puente, J. M. García Moreno, C. Borrué, B. Solano Vila, M. Álvarez Sauco, L. Vela, S. Escalante, E. Cubo, F. Carrillo Padilla, J. C. Martínez Castrillo, P. Sánchez Alonso, M. G. Alonso Losada, N. López Ariztegui, I. Gastón, J. Kulisevsky, M. Blázquez Estrada, M. Seijo, J. Rúiz Martínez, C. Valero, M. Kurtis, O. de Fábregues, J. González Ardura, C. Ordás, L. López Díaz, P. Mir, P. Martinez-Martin, COPPADIS Study Group

**Affiliations:** ^1^CHUAC, Complejo Hospitalario Universitario de A Coruña, A Coruña, Spain; ^2^CHUF, Complejo Hospitalario Universitario de Ferrol, A Coruña, Spain; ^3^Unidad de Trastornos del Movimiento, Servicio de Neurología y Neurofisiología Clínica, Instituto de Biomedicina de Sevilla, Hospital Universitario Virgen del Rocío/CSIC/Universidad de Sevilla, Seville, Spain; ^4^CIBERNED (Centro de Investigación Biomédica en Red Enfermedades Neurodegenerativas), Madrid, Spain; ^5^Hospital Universitari Mutua de Terrassa, Terrassa, Spain; ^6^Hospital Clínic de Barcelona, Barcelona, Spain; ^7^Centro Neurológico Oms 42, Palma de Mallorca, Spain; ^8^Consorci Sanitari Integral, Hospital Moisés Broggi, Sant Joan Despí, Barcelona, Spain; ^9^Hospital Universitario Son Espases, Palma de Mallorca, Spain; ^10^Hospital Universitario Vall d´Hebron, Barcelona, Spain; ^11^Complejo Hospitalario Universitario de Pontevedra (CHOP), Pontevedra, Spain; ^12^Hospital Universitario La Princesa, Madrid, Spain; ^13^Hospital Universitario Marqués de Valdecilla, Santander, Spain; ^14^Consorci Sanitari Integral, Hospital General de L´Hospitalet, L´Hospitalet de Llobregat, Barcelona, Spain; ^15^Hospital Universitario Clínico San Carlos, Madrid, Spain; ^16^Hospital Da Costa, Burela, Lugo, Spain; ^17^Hospital del Mar, Barcelona, Spain; ^18^Hospital Universitario Virgen Macarena, Sevilla, Spain; ^19^Hospital Infanta Sofía, Madrid, Spain; ^20^Institut d'Assistència Sanitària (IAS) - Institut Català de la Salut, Girona, Spain; ^21^Hospital General Universitario de Elche, Elche, Spain; ^22^Fundación Hospital de Alcorcón, Madrid, Spain; ^23^Hospital de Tortosa Verge de la Cinta (HTVC), Tortosa, Tarragona, Spain; ^24^Complejo Asistencial Universitario de Burgos, Burgos, Spain; ^25^Hospital Universitario de Canarias, San Cristóbal de la Laguna, Santa Cruz de Tenerife, Spain; ^26^Hospital Universitario Ramón y Cajal, Madrid, Spain; ^27^Hospital Universitario Puerta de Hierro, Madrid, Spain; ^28^Hospital Álvaro Cunqueiro, Complejo Hospitalario Universitario de Vigo (CHUVI), Vigo, Spain; ^29^Complejo Hospitalario de Toledo, Toledo, Spain; ^30^Complejo Hospitalario de Navarra, Pamplona, Spain; ^31^Hospital de Sant Pau, Barcelona, Spain; ^32^Hospital Universitario Central de Asturias, Oviedo, Spain; ^33^Hospital Universitario Donostia, San Sebastián, Spain; ^34^Hospital Arnau de Vilanova, Valencia, Spain; ^35^Hospital Ruber Internacional, Madrid, Spain; ^36^Hospital Universitario Lucus Augusti (HULA), Lugo, Spain; ^37^Hospital Rey Juan Carlos, Madrid, Spain; ^38^Complejo Hospitalario Universitario de Orense (CHUO), Orense, Spain; ^39^Fundación Curemos el Parkinson, C/Juana de Vega 23 2°, A Coruña 15004, Spain

## Abstract

**Introduction:**

In a degenerative disorder such as Parkinson's disease (PD), it is important to establish clinical stages that allow to know the course of the disease. Our aim was to analyze whether a scale combining Hoehn and Yahr's motor stage (H&Y) and the nonmotor symptoms burden (NMSB) (assessed by the nonmotor symptoms scale (NMSS)) provides information about the disability and the patient's quality of life (QoL) with regard to a defined clinical stage.

**Materials and Methods:**

Cross-sectional study in which 603 PD patients from the COPPADIS cohort were classified according to H&Y (1, stage I; 2, stage II; 3, stage III; 4, stage IV/V) and NMSB (A: NMSS = 0–20; B: NMSS = 21–40; C: NMSS = 41–70; D: NMSS ≥ 71) in 16 stages (HY.NMSB, from 1A to 4D). QoL was assessed with the PDQ-39SI, PQ-10, and EUROHIS-QOL8 and disability with the Schwab&England ADL (Activities of Daily Living) scale.

**Results:**

A worse QoL and greater disability were observed at a higher stage of H&Y and NMSB (*p* < 0.0001). Combining both (HY.NMSB), patients in stages 1C and 1D and 2C and 2D had significantly worse QoL and/or less autonomy for ADL than those in stages 2A and 2B and 3A and 3B, respectively (*p* < 0.005; e.g., PDQ-39SI in 1D [*n* = 15] vs 2A [*n* = 101]: 28.6 ± 17.1 vs 7.9 ± 5.8; *p* < 0.0001).

**Conclusion:**

The HY.NMSB scale is simple and reflects the degree of patient involvement more accurately than the H&Y. Patients with a lower H&Y stage may be more affected if they have a greater NMS burden.

## 1. Introduction

Parkinson's disease (PD) is a progressive neurodegenerative disorder causing motor and nonmotor symptoms (NMS) that result in disability, loss of patient autonomy, and caregiver burden [1]. In a degenerative disease, it is important to establish clinical stages that allow the determination of disease progression for a patient based on different specific symptoms. Ideally, this clinical graduation should be simple to carry out so that it can be used universally in clinical practice. In the case of PD, and based on the classic motor symptoms of the disease, the Hoehn and Yahr (H&Y) scale is used to describe the progression of PD [2]. The scale was originally described in 1967 and included stages 1 through 5. It has since been modified with the addition of stages 1.5 and 2.5 to help describe the intermediate course of the disease [3]. This rating system has been largely supplemented by, firstly, the Unified Parkinson's Disease Rating Scale (UPDRS) [4], and more recently, the MDS-Unified Parkinson's Disease Rating Scale (MDS-UPDRS) [5], which assess limitation of Activities of Daily Living (ADL) and NMS. However, evaluating the patient using the UPDRS and/or MDS-UPDRS takes time; specialization is required and, importantly, do not allow the patient to be classified into a clearly differentiated stage, and several NMS are not included. Validated tools for assessing NMS such as the NMSQuest [6] and the nonmotor symptoms scale (NMSS) [7] are used both in trials and in clinical practice. Furthermore, it has been demonstrated that NMS are an important determinant and deteriorating factor of the quality of life (QoL) of PD patients [8, 9]. Not only motor symptoms but also NMS increase in their severity and burden over time, increasing patients' disability, with additional worsening of their QoL, as well as caregivers' burden and consequential consumption of social resources by increasing societal costs. That is why for staging PD it would be necessary to combine a motor with a nonmotor scale, which would allow the patient to be classified into stages considering both the degree of motor and nonmotor involvement.

Recently, it has been suggested that gradation of PD according to the motor impairment and burden of NMS is an unmet need for an appropriate management of patients [10]. Ray Chaudhuri et al. proposed a PD classification by H&Y staging and NMS burden level and demonstrated a correlation of both H&Y staging and NMS burden to disability and QoL [11]. However, QoL and autonomy for ADL regarding the stage considering both together, motor and nonmotor stages, were not analyzed. The H&Y scale provides quick information about the patient's condition, but since it does not include NMS, it is not very sensitive to reflect the real impact of that condition. Our hypothesis is that a patient with a lower H&Y stage but a greater NMS burden may present a worse QoL and greater disability than another patient with a more advanced H&Y stage but a lower NMS burden, so it would be beneficial to combine both aspects on a scale. The aim of this study was to classify PD patients from the COPPADIS cohort [12, 13], regarding H&Y and NMS burden combined in a specific scale (HY.NMSB), and to compare QoL and autonomy for ADL between patients in a different HY.NMSB stages.

## 2. Materials and Methods

PD patients recruited from 35 centers of Spain from the COPPADIS cohort [13] from January 2016 to November 2017 were included in the study. Methodology about COPPADIS-2015 study can be consulted in https://bmcneurol.biomedcentral.com/articles/10.1186/s12883-016-0548-9.

This is a multicenter, observational, longitudinal-prospective, 5-year follow-up study designed to analyze disease progression in a Spanish population of PD patients. The data for the present study (cross-sectional study) were obtained from the baseline evaluation. All patients included were diagnosed according to UK PD Brain Bank criteria. Exclusion criteria were as follows: non-PD parkinsonism, dementia (Mini Mental State Examination (MMSE) < 26), age <18 or >75 years, inability to read or understand the questionnaires, to be receiving any advanced therapy (continuous infusion of levodopa or apomorphine and/or with deep brain stimulation), and the presence of comorbidity, sequelae, or any disorder that could interfere with the assessment.

Information on sociodemographic aspects, factors related to PD, comorbidity, and treatment was collected. Motor and NMS were evaluated using different validated scales [12]. In patients with motor fluctuations, the motor assessment (H&Y and UPDRS) was conducted during the OFF state (without medication in the last 12 hours; H&Y-OFF and UPDRS-III-OFF) and during the ON state (H&Y-ON and UPDRS-III-ON). However, in patients without motor fluctuations, it was only performed without medication (first thing in the morning without taking medication in the previous 12 hours). Moreover, in PD patients with motor fluctuations, the nonmotor assessment was conducted during the ON state [12]. The NMSS [7] was used for assessing NMS. This includes 30 items, each with a different nonmotor symptom. The symptoms refer to the 4 weeks prior to assessment. The total score for each item is the result of multiplying the frequency (0, never; 1, rarely; 2, often; 3, frequent; 4, very often) x severity (1, mild; 2, moderate; 3, severe) and will vary from 0 to 12 points. The scale score ranges from 0 to 360 points. The items are grouped into 9 different domains: (1) cardiovascular (items 1 and 2; score, 0 to 24); (2) sleep/fatigue (items 3, 4, 5, and 6; score, 0 to 48); (3) depression/apathy (items 7, 8, 9, 10, 11, and 12; score, 0 to 72); (4) perceptual problems/hallucinations (items 13, 14, and 15; score, 0 to 36); (5) attention/memory (items 16, 17, and 18; score, 0 to 36); (6) gastrointestinal tract (items 19, 20, and 21; score, 0 to 36); (7) urinary symptoms (items 22, 23, and 24; score, 0 to 36); (8) sexual dysfunction (items 25 and 26; score, 0 to 24); (9) miscellaneous (items 27, 28, 29, and 30; score, 0 to 48).

Three different instruments were used to assess QoL: (1) the PDQ-39 [14]; (2) a rating of global perceived QoL (PQ-10) on a scale from 0 (worst) to 10 (best) [8, 15]; and (3) the EUROHIS-QOL8 [16]. The PDQ-39 is a PD-specific questionnaire that assesses the patients' health-related QoL. There are 39 items grouped into 8 domains: (1) mobility (items 1 to 10); (2) Activities of Daily Living (items 11 to 16); (3) emotional well-being (items 17 to 22); (4) stigma (items 23 to 26); (5) social support (items 27 to 29); (6) cognition (items 30 to 33); (7) communication (items 34 to 36); and (8) pain and discomfort (items 37 to 39). For each item, the score may range from 0 (never) to 4 (always). The symptoms refer to the 4 weeks prior to assessment. Domain total scores are expressed as a percentage of the corresponding maximum possible score, and a Summary Index is obtained as average of the domain scores. The EUROHIS-QOL8 is an 8-item global QoL questionnaire (quality of life, health status, energy, autonomy for Activities of Daily Living, self-esteem, social relationships, economic capacity, and habitat) derived from the WHOQOL-BREF. For each item, the score ranges from 0 (not at all) to 5 (completely). The total score is expressed as the mean of the individual scores. A higher score indicates a better QoL. The Schwab and England Activities of Daily Living Scale (ADLS) was used for assessing disability [17]. Functional dependency was defined as an ADLS score less than 80% (80% = completely independent; 70% = not completely independent) [18].

### 2.1. Data analysis

Data were processed using SPSS 20.0 for Windows. NMS burden was defined as follows: mild (NMSS 1-20); moderate (NMSS 21-40); severe (NMSS 41-70); and very severe (NMSS > 70) [10]. Each domain of the NMSS was expressed as a percentage: (score/total score) × 100. The patients were classified according to H&Y-OFF (1, stage I; 2, stage II; 3, stage III; 4, stage IV /V) and NMS burden (A: 0-20; B: 21-40; C: 41-70; D: ≥ 71) in 16 stages (HY.NMSB): 1A, 1B, 1C, 1D, 2A, 2B, 2C, 2D, 3A, 3B, 3C, 3D, 4A, 4B, 4C, and 4D. PDQ-39 was expressed as a Summary Index (PDQ-39SI): (score/156) × 100. For comparisons between patients with a different H&Y stage, NMS burden stage, and/or HY.NMSB stage, chi-squared, ANOVA, and/or Mann–Whitney–Wilcoxon test were applied. With the aim of determining if the HY.NMSB contributes to the patient's QoL independently of other factors, a multiple regression analysis was conducted (PDQ-39SI as dependent variable). A *p* value < 0.05 was considered significant.

### 2.2. Standard Protocol Approvals, Registrations, and Patient Consent

For this study, we received approval from the *Comité de Ética de la Investigación Clínica de Galicia* from Spain (2014/534; 02/DEC/2014). Written informed consents from all participants in this study were obtained before the start of the study. COPPADIS-2015 was classified by the AEMPS (*Agencia Española del Medicamento y Productos Sanitarios*) as a postauthorization prospective follow-up study with the code COH-PAK-2014-01.

## 3. Results

**Figure 1:**
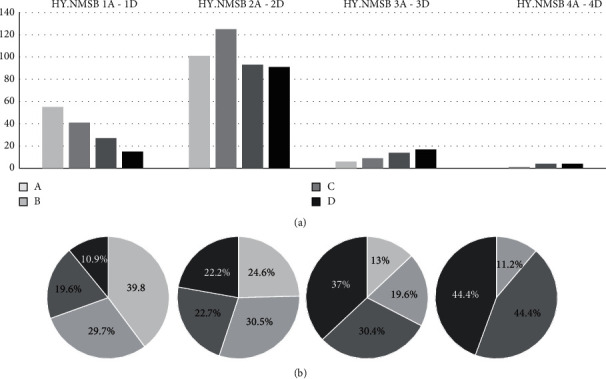
Total number (a) and percentage (b) of PD patients presenting with different stages of the HY.NMSB scale (from 1A to 4D) (*n *=* *603).

A total of 603 PD patients (62.7 ± 8.9 years old; 59.5% males) from the COPPADIS cohort were included in the analysis. The mean disease duration was 5.7 ± 4.5 years. One-hundred and twenty-eight (22.9%) patients were in stage I of H&Y, 407 (67.5%) in stage II of H&Y, 49 (8.1%) in stage III of H&Y, and only 9 (1.5%) in stage IV/V of H&Y. The mean NMSS total score was 46.7 ± 38.2, presenting 162 (26.9%) patients with mild NMS burden, 174 (28.8%) with moderate NMS burden, 140 (23.2%) with severe NMS burden, and 127 (21.1%) with very severe NMS burden. No patient presented absence of nonmotor symptoms (NMSS = 0). Data about PD-related variables are shown in Table SM 1. When H&Y and NMS burden were combined (HY.NMSB), a higher percentage of patients with severe or very severe NMS burden in advanced H&Y stages (III and/or IV/V) (*p* < 0.0001) was observed (Figure 1).

**Figure 2:**
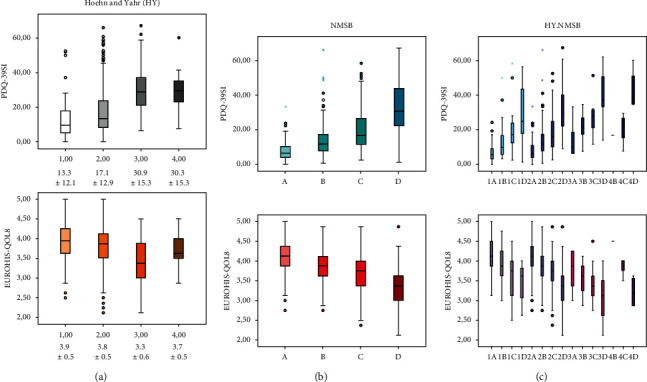
PDQ-39SI and EUROHIS-QOL8 scores in patients with regard to the H&Y (a), the nonmotor symptoms burden (NMSB) (b), and both (HY.NMSB) (c) (*n* = 603).

A worse QoL and a greater disability were associated with a higher H&Y stage. Specifically, the PDQ-39SI and the EUROHIS-QOL8 total score were significantly lower and higher, respectively, in patients with a lower H&Y (Table 1 and Figure 2(a)). The ADLS score was higher (indicative of lower disability) in patients with a lower H&Y (Table 1). When patients with a consecutive stage of H&Y were compared, the most significant differences were observed between patients with a stage II of H&Y and those ones with stage III, but no differences were observed between patients with a stage III of H&Y and those ones with stage IV (only 9 patients in this last subgroup) (Table 1). QoL and disability were related to NMS burden as well, so the higher the NMS burden stage, the worse the QoL, and the greater the disability (Table 2 and Figure 2(b)). After classifying participants by combining both scales, H&Y and NMSS (NMSB), QoL and disability were related to the HY.NMSB stage (Figure 2(c)): PDQ-39SI, from 6.7 ± 4.9 (HY.NMSB 1A) to 42.9 ± 11.9 (HY.NMSB 4D) (*p* < 0.0001); EUROHIS-QOL8 total score, from 4.1 ± 0.5 (HY.NMSB 1A) to 3.1 ± 0.6 (HY.NMSB 3D) (*p* < 0.0001; only 1 patient in the stage 4B but with a score of 4.5); and ADLS score, from 94.9 ± 5.7 (HY.NMSB 1A) to 55 ± 19.1 (HY.NMSB 4D) (*p* < 0.0001). With regard to our hypothesis, it was observed that patients with a lower stage of H&Y could have a worse QoL and/or a greater disability if they had a greater NMS burden (Tables 3 and 4). For example, patients with stage I of H&Y and very severe NMS burden (HY.NMSB 1D; *n* = 15) compared to patients with stage II of H&Y but mild NMS burden (HY.NMSB 2A; *n* = 101) had a higher PDQ-39SI (28.6 ± 17.1 vs 7.9 ± 5.8; *p* < 0.0001) and a lower PQ-10 (6.4 ± 1.5 vs 7.9 ± 1.2; *p* < 0.0001), EUROHIS-QOL8 (3.5 ± 0.4 vs 4.1 ± 0.4*p* < 0.0001), and ADLS score (88 ± 6.8 vs 91.8 ± 5.9; *p*=0.025) (Table 3 and Figure 2). Even PDQ-39SI (198 ± 11.9 vs 13.8 ± 9.8; *p*=0.003) and EUROHIS-QOL8 score (3.6 ± 0.5 vs 3.9 ± 0.5; *p*=0.030), we are significantly higher and lower, respectively, in those patients with stage I of H&Y and severe NMS burden HY.NMSB 1C; *n* = 27) than those in ones with stage < II of H&Y and moderate NMS burden (HY.NMS burden 2B; *n* = 125) (Table 3 and Figure 2). When patients with a stage II of H&Y were compared with those ones with a stage III, a worse QoL was observed in patients with stage II and very severe NMS burden (HY.NMSB 2D; *n* = 91) than those in patients with a stage III of H&Y but mild NMS burden (HY.NMSB 3A; *n* = 6) or moderate NMS burde*n* = (HY.NMSB 3B; *n* = 9): PDQ-39SI 31.8 ± 3.8 vs 14.2 ± 10.9 *p*=0.003; 31.8 ±13.8 vs. 21.5 ± 7.9 (*p*=0.029): PQ-10, 6.2 ± 1.6 vs 8.5 ± 1.5 (*p*=0.003); EUROHIS-QOL8, 3.8 ± 0.6 vs 3.6 ± 0.4 (*p*=0.048) (Table 4 and Figure 2).

In a simple linear regression model, the HY.NMSB scale predicted the PDQ-39SI: *β* = 0.480; CI 95%, 1.981 – 2.661; *p* < 0.0001. After adjustment to other covariates (age, gender, disease duration, levodopa equivalent daily dose, UPDRS-IV, FOGQ, and BDI-II), the HY.NMSB stage contributed significantly to the patient's QoL (PDQ-39SI as dependent variable) as well: adjusted *R*-squared 0.591; *β* = 0.089; CI 95%, 0.098 – 0.770; *p*=0.011 (Table 2. SM). As compared to the classical H&Y stage alone (not significant in the model), the HY.NMS was multiplied by 12.7 the size effect over the PDQ-39SI (*β* standardized coefficient of 0.007 for the H&Y in a model with age, gender, disease duration, levodopa equivalent daily dose, UPDRS-IV, FOGQ, BDI-II, and NMSS (*p*=0.823) vs 0.089 for the HY.NMS in the model with the same covariates included except the NMSS (*p*=0.011)).

## 4. Discussion

The present study observed that the use in PD patients of a scale that combines the H&Y stage with the NMSS (HY.NMSB) could be useful since it would not only inform about motor and nonmotor aspects but would also serve to know how is the patient's QoL and autonomy for ADL. This is relevant because many PD patients can be in stages I to III of H&Y for many years and stratification regarding NMS burden providing useful information not only for diagnosis but also for monitoring the outcome and ideally the response to a medication.

Ray Chaudhuri et al. [11] proposed a new strategy for clinical classification of PD patients using the NMSS in 5 stratified levels of burden (0 = no NMS; 1 = NMSS, 1-20; 2 = NMSS, 21-40; 3 = NMSS, 41-70; 4 = NMSS > 70) and suggested that this simple assessment could be added to existing motor-based staging (i.e., H & Y) to complement PD assessment and avoid overlooking the weight of the NMS. In 951 PD patients, these authors observed a significant influence of NMS burden on disability and QoL, highlighting the need to include an NMS evaluation for a complete assessment of PD patients. We observed the same in 603 PD patients from the COPPADIS cohort. However, here, we define specifically a scale (HY.NMSB) combining the H&Y stage with the NMS burden: firstly, a number for the H&Y from 1 (stage I) to 4 (stage IV/V); secondly, a letter for the NMS burden from A (non NMS or mild NMS burden; NMSS 0-20) to D (very severe burden; NMSS > 70). Combining the number with the letter, a total of 16 stages are defined, from HY.NMSB 1A (H&Y I and non-NMS/mild NMS burden) to 4D (H&Y IV/V and very severe NMS burden). PD patients without NMS (i.e., NMSS total score = 0) are rare (none in this cohort), but in any case, they are included as “A” because there is really no difference between, for example, a patient with NMSS total score = 0 and another one with NMSS total score = 1 or 3. So, “A” is defined as a patient without NMS or mild NMS burden. On the other hand and with the idea of simplifying the scale, very advanced PD patients with regard to motor stage (H&Y IV and V) are considered together as number 4. After applying this scale (HY.NMSB) for the first time, we observed that QoL and disability were related to H&Y but NMS burden as well, so patients with a lower H&Y but a greater NMS burden can perceive a worse QoL and greater disability than patients with a higher H&Y stage but lower NMS burden. Conventionally, H&Y stages I and II represent mild PD, but this qualification cannot be supported attending the load of NMS and any domain/s they belong. The NMS present in PD may be very variable in number and type, and they maintain only a moderate association with the motor disturbances [10, 11, 19, 20]. In fact, although as expected, patients with mild NMS burden (*A*; 39.8%) were the most frequent in the group with a stage I of H&Y and patients with very severe NMS burden (*D*; 44.4%) in the group with H&Y IV/V; more than 30% of the patients in stage I of H&Y had severe or very severe NMS burden. Clinical and neuropathological data are now emerging supporting the concept of the nonmotor dominant endophenotype [21], and it seems necessary in daily practice to know the frequency and the severity of NMS in PD patients, even in early PD patients, because NMS burden could be significant, and this one impacts on their QoL and contributes to disability [7, 9, 15, 22]. Very recently, two PD subtypes have been suggested [23, 24], and it would be of great interest to know if very early PD patients with very severe NMS burden could correspond with the body-first (bottom-up) type and those with mild NMS burden with the brain-first (top-down) type.

The application of the HY.NMSB scale could have different uses: (1) a fast and relatively simple way of knowing the motor and nonmotor states of a PD patient, stratifying him/her into a group (diagnosis value; first visit); (2) to monitor the long-term evolution of the patient (prognosis value; follow-up visits); (3) to monitor the response of a patient to a specific therapeutic intervention. In fact, the NMSS total score has been considered as the primary efficacy variable in recent trials [25], and it is known that some NMS can be improved, with dopaminergic medication or nondopaminergic medication [26]. In this context, the HY.NMSB could be used for defining a specific population or as an outcome parameter in clinical trials. For example, nabilone has very recently demonstrated to improve NMS in PD patients in a phase 2 trial [27], being an interesting possibility to identify what patients changed from a superior stage of the HY.NMSB to an inferior stage (e.g., from 2C to 2 B). Finally, the HY-NMSB scale could be useful to indirectly estimate the patient's perception of QoL and disability. The correlation of H&Y, NMSS, and NMS burden with QoL and disability has been frequently reported [7–9, 22], including in PD patients from the COPPADIS cohort [15, 18], but this is the first time that the relationship considering both motor stage (H&Y) and NMS burden (NMSS) at the same time has been analyzed, and it is important because the influence of NMS burden on QoL perception is critical. An inherent limitation of the proposed classification (HY.NMSB) is the fact that the classification according to NMS is carried out taking into account the total NMS burden but without considering what exactly these symptoms are. Importantly, some NMS could help clinical practitioners to identify patients who are at different stages of the disease, such as hallucinations, fainting, inability to control body sphincters, or believing in unlikely facts [28]. Moreover, and compared with the International Parkinson and Movement Disorder Society─Nonmotor Rating Scale (MDS-NMS) [29], the NMSS collects the patient's perception about different NMS in the previous 4 weeks but does not about nonmotor fluctuations.

A very important limitation is that our sample is not fully representative of the PD population due to inclusion and exclusion criteria (i.e., age limit, no dementia, no severe comorbidities, and no second line therapies) which subsequently entails a bias toward early PD. The majority of the patients from this cohort were in the stage I or II of the H&Y (90.4%), so the same analysis with the proposed classification should be carried out in a cohort with more patients in advanced stages of H&Y. In spite of this and importantly, during the first 5 to 10 years of the disease, many patients with PD will be in stage II of the H&Y, and introducing the NMS burden will help to differentiate the degree of nonmotor affectation, that importantly correlates with QoL perception. In other words, the results of the present study are applicable for a long time to the majority of PD patients, especially in early young PD patients. Second, all scales or questionnaires used for assessing motor and NMS are validated except PQ-10 [8, 15]. Third, NMS were recorded with the NMSS, but specifically, as we commented nonmotor fluctuations were not identified [30]. Fourth, the OFF state (12 hours without taking medication) was considered for defining the H&Y stage because it represents a more natural state of the disease less conditioned by the symptomatic effect of the medication. Moreover, in PD patients with motor fluctuations, the symptoms during the OFF state mostly impact on QoL and autonomy. In any case, previously, similar results applying the HY.NMSB were observed when the H&Y stage was defined during the ON state in 149 PD patients from the CASINO cohort [8, 31]. In the COPPADIS cohort, the results were similar as well when the H&Y was defined during the ON state in those PD patients with motor fluctuations (data not shown). Fifth, the time it took to administer the HY.BMSB scale was not calculated. Finally, this is a cross-sectional study, but the aim of the COPPADIS-2015 study [12] is to follow-up the cohort for 5 years, so changes in HY.NMSB and the relationship with changes in other variables could be analyzed.

In conclusion, this is the first time that a specific scale combing the H&Y stage and the NMSS (HY.NMSB scale) is applied in PD patients for knowing the relationship with the patient's QoL perception and disability regarding the stage. The HY.NMSB scale is simple and reflects the degree of patient involvement more accurately than the H&Y. Patients with a lower H&Y stage may be more affected if they have a greater NMS burden. These results need to be replicated in a larger and well-distributed cohort of patients by motor stage.

## Figures and Tables

**Table 1:** Quality of life (PDQ-39SI and EUROHIS-QOL8) and disability (ADLS score) in PD patients with regard to Hoehn and Yahr stage (*s* = 603).

	H&Y 1, *N* = 138	H&Y 2, *N* = 407	H&Y 3, *N* = 49	H&Y 4, *N* = 9	*p* ^*a*^	*p* ^*b*^	*p* ^*c*^	*p* ^*d*^
PDQ-39SI	13.3 ± 12.1	17.1 ± 12.9	30.9 ± 15.3	30.3 ± 15.3	**<0.0001**	**0.037**	**<0.0001**	0.908
Mobility	10.9 ± 14.2	16.1 ± 18	41.6 ± 23.8	37.2 ± 24.3	**<0.0001**	**0.036**	**<0.0001**	0.613
Activities of Daily Living	13.4 ± 14.3	18.3 ± 18.1	33.6 ± 24	29.1 ± 19.1	**<0.0001**	**0.023**	**<0.0001**	0.604
Emotional well-being	18.9 ± 16.9	21.6 ± 20.7	34.3 ± 21.9	32.8 ± 13.2	**<0.0001**	0.372	**<0.0001**	0.846
Stigma	12.3 ± 17	13.2 ± 19.7	16.1 ± 22.1	22.9 ± 32.7	0.316	0.839	0.341	0.432
Social support	6.8 ± 14.3	8.6 ± 16.9	9.7 ± 19.7	12.9 ± 22.1	0.497	0.492	0.686	0.656
Cognition	14.9 ± 16.4	20.1 ± 18.1	27.2 ± 18.9	31.2 ± 17.9	**<0.0001**	**0.015**	**0.011**	0.553
Communication	8.8 ± 13.8	9.5 ± 14.2	17.3 ± 19.7	19.4 ± 17.6	**0.001**	0.522	**0.001**	0.766
Pain and discomfort	21.1 ± 19.9	26.9 ± 22.9	42.5 ± 22.5	40.7 ± 28.9	**<0.0001**	0.065	**<0.0001**	0.835
PQ-10	7.5 ± 1.5	7.2 ± 6.2	6.2 ± 2.1	6.9 ± 1.4	**<0.0001**	0.234	**<0.0001**	0.375
EUROHIS-QOL8	3.9 ± 0.5	3.8 ± 0.5	3.3 ± 0.6	3.7 ± 0.5	**<0.0001**	0.117	**<0.0001**	0.135
Quality of life	3.9 ± 0.7	3.8 ± 0.7	3.2 ± 0.8	3.8 ± 0.4	**<0.0001**	0.207	**<0.0001**	0.063
Health status	3.4 ± 0.8	3.1 ± 0.9	2.5 ± 0.9	2.9 ± 0.8	**<0.0001**	**0.012**	**<0.0001**	0.296
Energy	3.9 ± 0.8	3.7 ± 0.8	3.2 ± 0.8	3.7 ± 1.1	**<0.0001**	0.195	**<0.0001**	0.156
Autonomy for ADL	3.8 ± 0.7	3.6 ± 0.8	2.8 ± 0.8	3.1 ± 0.8	**<0.0001**	**0.023**	**<0.0001**	0.289
Self-esteem	3.9 ± 0.7	3.8 ± 0.8	3.4 ± 0.9	3.4 ± 0.7	**0.001**	0.437	**0.001**	0.863
Social relationships	4 ± 0.7	4.1 ± 0.7	3.7 ± 0.8	4 ± 0.9	**0.021**	0.890	**0.004**	0.388
Economic capacity	3.9 ± 0.7	3.7 ± 0.8	3.7 ± 1	3.9 ± 0.7	0.414	0.819	0.160	0.546
Habitat	4.3 ± 0.7	4.2 ± 0.7	4.1 ± 0.8	4.4 ± 0.5	0.287	0.832	0.112	0.161
ADLS score	93.5 ± 6.9	87.8 ± 9.4	77.1 ± 13.1	72.5 ± 23.8	**<0.0001**	**<0.0001**	**<0.0001**	0.416
Functional dependency (%)	0.7	8.8	42.9	37.5	**<0.0001**	**<0.0001**	**<0.0001**	0.546

Chi-squared, Mann–Whitney–Wilcoxon, and ANOVA test were applied. The results represent percentages or mean±SD; *p*^*a*^, all groups; *p*^*b*^, H&Y 2 vs H&Y 1; *p*^*c*^, H&Y 3 vs H&Y 2; *p*^*d*^, H&Y 4 vs H&Y 3. ADL, Activities of Daily Living; ADLS, Schwab and England Activities of Daily Living Scale; EUROHIS-QOL8, EUROHIS-QOL 8-item index; H&Y, Hoehn and Yahr; PDQ-39SI, 39-item Parkinson's Disease Quality of Life Questionnaire Summary Index.

**Table 2:** Quality of life (PDQ-39SI and EUROHIS-QOL8) and disability (ADLS score) in PD patients with regard to nonmotor symptoms burden: mild (NMS 1-20); moderate (NMS total score 21-40); severe (NMS total score 41-70); very severe (NMS total score > 70).

	Mild, *N *=* *162	Moderate, *N *=* *174	Severe, *N *=* *140	Very severe, *N *=* *127	*p* ^*a*^	*p* ^*b*^	*p* ^*c*^	*p* ^*d*^
PDQ-39SI	7.7 ± 5.7	13.8 ± 9.7	19.9 ± 10.7	32.9 ± 14.4	**<0.0001**	**<0.0001**	**<0.0001**	**<0.0001**
Mobility	6.2 ± 11.5	12.7 ± 15.6	19.7 ± 15.9	35.3 ± 22.9	**<0.0001**	**<0.0001**	**0.029**	**<0.0001**
Activities of Daily Living	9.5 ± 11.1	15.8 ± 15.4	19.8 ± 16.7	32.4 ± 23.3	**<0.0001**	**<0.0001**	**<0.0001**	**<0.0001**
Emotional well-being	9.9 ± 10.8	15.6 ± 13.9	26.6 ± 18.7	41.6 ± 22.6	**<0.0001**	**0.001**	0.149	**<0.0001**
Stigma	7.6 ± 14.1	13.7 ± 18.9	10.8 ±16.2	22.9 ± 25.5	**<0.0001**	**0.025**	**0.001**	**<0.0001**
Social support	2.2 ± 8.9	4.9 ± 12.8	10.6 ± 17.5	18.6 ± 21.9	**<0.0001**	**<0.0001**	**<0.0001**	**0.001**
Cognition	7.2 ± 9.6	16.2 ± 13.9	24.6 ± 16.2	34.9 ± 20.1	**<0.0001**	**<0.0001**	**0.036**	**<0.0001**
Communication	3.5 ± 7.3	8.3 ± 12.4	11.6 ± 14.7	19.3 ± 19.7	**<0.0001**	**<0.0001**	**<0.0001**	**<0.0001**
Pain and discomfort	14.7 ± 15.6	20.7 ± 18.9	30.7 ± 19.6	47.4 ± 24.8	**<0.0001**	**0.002**	**<0.0001**	**<0.0001**
PQ-10	7.9 ± 1.2	7.5 ± 1.4	6.9 ± 1.4	6.1 ± 1.7	**<0.0001**	**<0.0001**	**0.003**	**<0.0001**
EUROHIS-QOL8	4.1 ± 0.5	3.9 ± 0.4	3.6 ± 0.5	3.3 ± 0.6	**<0.0001**	**<0.0001**	**<0.0001**	**<0.0001**
Quality of life	4.1 ± 0.5	3.9 ± 0.6	3.7 ± 0.7	3.3 ± 0.8	**<0.0001**	**0.005**	**0.001**	**<0.0001**
Health status	3.5 ± 0.8	3.3 ± 0.8	3 ± 0.8	2.6 ± 0.9	**<0.0001**	**0.001**	**0.006**	**<0.0001**
Energy	4.2 ± 0.7	3.8 ± 0.7	3.6 ± 0.8	3.2 ± 0.9	**<0.0001**	**<0.0001**	**0.004**	**<0.0001**
Autonomy for ADL	4 ± 0.7	3.8 ± 0.8	3.4 ± 0.8	3 ± 0.9	**<0.0001**	**0.001**	**<0.0001**	**<0.0001**
Self-esteem	4.2 ± 0.6	3.9 ± 0.7	3.6 ± 0.7	3.3 ± 0.9	**<0.0001**	**0.010**	**<0.0001**	**0.001**
Social relationships	4.4 ± 0.6	4.1 ± 0.6	3.9 ± 0.7	3.6 ± 0.8	**<0.0001**	**<0.0001**	0.059	**<0.0001**
Economic capacity	4.1 ± 0.8	3.9 ± 0.7	3.8 ± 0.7	3.5 ± 0.9	**<0.0001**	0.079	0.180	**0.001**
Habitat	4.4 ± 0.7	4.3 ± 0.6	4.2 ± 0.7	3.9 ± 0.7	**<0.0001**	0.105	0.123	**0.011**
ADLS score	92.9 ± 6.1	90.2 ± 8.3	86.5 ± 10.4	80.5 ± 12.9	**<0.0001**	**0.001**	**<0.0001**	**<0.0001**
Functional dependency (%)	0.6	4.6	11.5	28.3	**<0.0001**	**0.024**	**0.019**	**<0.0001**

**Table 3:** Quality of life (PDQ-39SI and EUROHIS-QOL8) and disability (ADLS score) in patients with stages 1C, 1D, 2A, or 2B of the HY-NMSB scale.

	1C, *N* = 27	1D, *N* = 15	2A, *N* = 101	2B, *N* = 125	*p* ^*a*^	*p* ^*b*^	*p* ^*c*^	*p* ^*d*^
PDQ-39SI	19.8 ± 11.9	28.6 ± 17.1	7.9 ± 5.8	13.8 ± 9.8	**<0.0001**	**<0.0001**	**<0.0001**	**0.003**
Mobility	18.1 ± 14.9	27.8 ± 17.7	6.1 ± 10.5	13 ± 16.2	**<0.0001**	**0.001**	**<0.0001**	**0.035**
Activities of Daily Living	16.5 ± 15.2	28.3 ± 18.3	10.9 ± 12.4	15.9 ± 16.1	**<0.0001**	**0.007**	**0.056**	0.710
Emotional well-being	28.7 ± 16.4	35.5 ± 21.3	8.9 ± 9.4	14.9 ± 13.7	**<0.0001**	**<0.0001**	**<0.0001**	**<0.0001**
Stigma	16.4 ± 20.4	23.8 ± 23.9	8.4 ± 15.7	13.9 ± 19.9	**0.013**	0.140	**0.030**	0.448
Social support	13.6 ± 17.8	19.4 ± 22.6	2.4 ± 9.9	5.3 ± 13.8	**<0.0001**	**0.003**	**<0.0001**	**0.005**
Cognition	21.9 ± 17.4	32.5 ± 18.8	8.3 ± 9.6	15.9 ± 13.7	**<0.0001**	**0.001**	**<0.0001**	0.099
Communication	14.2 ± 15.9	20.6 ± 19.6	4.1 ± 8.4	7.9 ± 12.1	**<0.0001**	**0.007**	**<0.0001**	**0.026**
Pain and discomfort	27.8 ± 20.1	38.3 ± 27.4	13.5 ± 13.5	21.2 ± 20.3	**<0.0001**	**0.010**	**<0.0001**	0.053
PQ-10	6.9 ± 1.6	6.4 ± 1.5	7.9 ± 1.2	7.5 ± 1.5	**<0.0001**	**0.008**	**0.008**	0.201
EUROHIS-QOL8	3.6 ± 0.5	3.5 ± 0.4	4.1 ± 0.4	3.9 ± 0.5	**<0.0001**	**0.004**	**<0.0001**	**0.030**
Quality of life	3.6 ± 0.6	3.2 ± 0.6	4.1 ± 0.6	3.9 ± 0.6	**<0.0001**	**<0.0001**	**0.001**	**0.048**
Health status	3.2 ± 0.9	3.2 ± 0.6	3.6 ± 0.7	3.3 ± 0.8	**0.014**	0.726	0.051	0.888
Energy	3.4 ± 0.7	3.4 ± 0.6	4.1 ± 0.6	3.8 ± 0.7	**<0.0001**	**0.025**	**<0.0001**	**0.009**
Autonomy for ADL	3.5 ± 0.7	3.5 ± 0.5	4.1 ± 0.6	3.7 ± 0.7	**<0.0001**	0.065	**<0.0001**	0.089
Self-esteem	3.6 ± 0.8	3.5 ± 0.6	4.2 ± 0.6	3.9 ± 0.7	**<0.0001**	**0.015**	**<0.0001**	**0.016**
Social relationships	3.9 ± 0.6	3.8 ± 0.6	4.4 ± 0.6	4.2 ± 0.6	**0.001**	**0.023**	**<0.0001**	**0.022**
Economic capacity	3.7 ± 0.7	3.4 ± 0.7	3.9 ± 0.8	3.9 ± 0.6	**0.010**	**0.025**	**0.014**	0.054
Habitat	3.9 ± 0.8	3.9 ± 0.7	4.4 ± 0.6	4.3 ± 0.6	**0.005**	**0.001**	0.160	0.114
ADLS score	92.6 ± 7.1	88 ± 6.8	91.8 ± 5.9	89.5 ± 8	**0.025**	0.246	0.461	0.061
FD (%)	0	0	1	4	0.871	0.562	0.789	0.371

Chi-squared and Mann–Whitney–Wilcoxon test were applied. The results represent percentages or mean ± SD; *p*^*a*^, 1D vs 2A; *p*^*b*^, 1D vs 2B; *p*^*c*^, 1C vs 2A; *p*^*d*^, 1C vs 2B. ADLS, Schwab and England Activities of Daily Living Scale; EUROHIS-QOL8, EUROHIS-QOL 8-item index; PDQ-39SI, 39-item Parkinson's Disease Quality of Life Questionnaire Summary Index.

**Table 4:** Quality of life (PDQ-39SI and EUROHIS-QOL8) and disability (ADLS score) in patients with stages 2C, 2D, 3A, or 3B of the HY-NMSB scale.

	2C, *N* = 93	2D, *N* = 91	3A, *N* = 6	3B, *N* = 9	*p* ^*a*^	*p* ^*b*^	*p* ^*c*^	*p* ^*d*^
PDQ-39SI	18.5 ± 10.2	31.8 ± 13.8	14.2 ± 10.9	21.5 ± 7.9	**0.003**	**0.029**	0.173	0.238
Mobility	17.2 ± 14.9	32 ± 2.1	27.1 ± 31.2	27.5 ± 16.3	0.372	0.722	0.713	**0.048**
Activities of Daily Living	18.4 ± 16.4	31.1 ± 23.3	6.9 ± 7.7	25.9 ± 11.7	**0.005**	**0.722**	0.081	0.084
Emotional well-being	25.6 ± 20.1	41.7 ± 23.1	15.9 ± 20.8	24.5 ± 19.5	**0.014**	**0.036**	0.203	0.953
Stigma	9.3 ± 15.1	22.4 ± 24.4	7.3 ± 15	6.9 ± 14.1	0.109	**0.073**	0.636	0.749
Social support	9.9 ± 16.7	19.1 ± 21.9	0 ± 0	2.8 ± 4.2	**0.015**	**0.048**	0.081	0.526
Cognition	24.4 ± 15.9	35.7 ± 21.2	1 ± 2.6	24.3 ± 13.4	**<0.0001**	0.109	**<0.0001**	0.948
Communication	9.5 ± 13.6	18.4 ± 18.7	2.8 ± 6.8	13.9 ± 13.8	**0.025**	0.633	0.183	0.226
Pain and discomfort	30.9 ± 19.6	46.1 ± 24.6	34.7 ± 22.6	28.7 ± 19.6	0.209	**0.031**	0.830	0.673
PQ-10	7 ± 1.4	6.2 ± 1.6	8.5 ± 1.5	7.2 ± 1.3	**0.003**	0.069	**0.025**	0.620
EUROHIS-QOL8	3.7 ± 0.4	3.3 ± 0.6	3.8 ± 0.6	3.6 ± 0.4	**0.048**	0.169	0.590	0.377
Quality of life	3.7 ± 0.7	3.4 ± 0.7	4 ± 0.9	3.6 ± 0.5	0.108	0.563	0.462	0.328
Health status	3 ± 0.7	2.6 ± 0.9	3.2 ± 0.7	2.7 ± 0.5	0.134	0.774	0.598	0.139
Energy	3.7 ± 0.7	3.2 ± 0.9	3.8 ± 0.4	3.6 ± 0.5	0.078	0.293	0.553	0.544
Autonomy for ADL	3.4 ± 0.7	3 ± 0.9	3 ± 0.6	3.2 ± 0.7	0.819	0.625	0.124	0.340
Self-esteem	3.6 ± 0.7	3.2 ± 0.9	4 ± 0.9	3.8 ± 0.7	0.083	0.112	0.335	0.696
Social relationships	4 ± 0.7	3.6 ± 0.8	4 ± 0.7	3.9 ± 0.6	0.115	0.322	0.649	0.520
Economic capacity	3.9 ± 0.7	3.5 ± 0.9	4.2 ± 0.7	3.8 ± 1.1	0.064	0.231	0.311	0.855
Habitat	4.3 ± 0.6	3.9 ± 0.7	4.2 ± 0.7	4.1 ± 0.9	0.369	0.255	0.713	0.751
ADLS score	86.5 ± 9.3	81.8 ± 11.5	90 ± 8.9	83.3 ± 10	0.091	0.769	0.421	0.278
FD (%)	9.7	25.3	0	22.2	0.187	0.601	0.556	0.250

Chi-squared and Mann–Whitney–Wilcoxon test were applied. The results represent percentages or mean ± SD; *p*^*a*^, 2D vs 3A; *p*^*b*^, 2D vs 3B; *p*^*c*^, 2C vs 3A; *p*^*d*^, 2C vs 3B. ADLS, Schwab and England Activities of Daily Living Scale; EUROHIS-QOL8, EUROHIS-QOL 8-item index; PDQ-39SI, 39-item Parkinson's Disease Quality of Life Questionnaire Summary Index.

**Table 5:** COPPADIS study group.

Name (last name, first name)	Location	Role	Contribution
Astrid Adarmes, Daniela	Hospital Universitario Virgen del Rocío, Sevilla, Spain	Site investigator	Evaluation of participants and/or data management
Almeria, Marta	Hospital Universitari Mutua de Terrassa, Terrassa, Barcelona, Spain	Site investigator	Neuropsychologist; evaluation of participants
Alonso Losada, Maria Gema	Hospital Álvaro Cunqueiro, Complejo Hospitalario Universitario de Vigo (CHUVI), Vigo, Spain	Site investigator /PI	Coordination at the center Evaluation of participants and/or data management
Alonso Cánovas, Araceli	Hospital Universitario Ramón y Cajal, Madrid, Spain	Site investigator	Evaluation of participants and/or data management
Alonso Frech, Fernando	Hospital Universitario Clínico San Carlos, Madrid, Spain	Site investigator	Evaluation of participants and/or data management
Aneiros Díaz, Ángel	Complejo Hospitalario Universitario de Ferrol (CHUF), Ferrol, A Coruña, Spain	Site investigator /PI	Coordination at the center Evaluation of participants and/or data management
Álvarez, Ignacio	Hospital Universitari Mutua de Terrassa, Terrassa, Barcelona, Spain	Site investigator	Evaluation of participants and/or data management
Álvarez Sauco, María	Hospital General Universitario de Elche, Elche, Spain	Site investigator /PI	Coordination at the center Evaluation of participants and/or data management
Arnáiz, Sandra	Complejo Asistencial Universitario de Burgos, Burgos, Spain	Site investigator	Evaluation of participants and/or data management
Arribas, Sonia	Hospital Universitari Mutua de Terrassa, Terrassa, Barcelona, Spain	Site investigator	Neuropsychologist; evaluation of participants
Ascunce Vidondo, Arancha	Complejo Hospitalario de Navarra, Pamplona, Spain	Site investigator	Evaluation of participants and/or data management
Aguilar, Miquel	Hospital Universitari Mutua de Terrassa, Terrassa, Barcelona, Spain	Site investigator	Evaluation of participants and/or data management
Ávila Rivera, Maria Asunción	Consorci Sanitari Integral, Hospital General de L´Hospitalet, L´Hospitalet de Llobregat, Barcelona, Spain	Site investigator /PI	Coordination at the center Evaluation of participants and/or data management
Bernardo Lambrich, Noemí	Hospital de Tortosa Verge de la Cinta (HTVC), Tortosa, Tarragona, Spain	Site investigator	Evaluation of participants and/or data management
Bejr-Kasem, Helena	Hospital de Sant Pau, Barcelona, Spain	Site investigator	Evaluation of participants and/or data management
Blázquez Estrada, Marta	Hospital Universitario Central de Asturias, Oviedo, Spain	Site investigator	Evaluation of participants
Botí González, Maria Ángeles	Hospital Universitari Mutua de Terrassa, Terrassa, Barcelona, Spain	Site investigator	Neuropsychologist; evaluation of participants
Borrué, Carmen	Hospital Infanta Sofía, Madrid, Spain	Site investigator /PI	Coordination at the center Evaluation of participants and/or data management
Buongiorno, Maria Teresa	Hospital Universitari Mutua de Terrassa, Terrassa, Barcelona, Spain	Site investigator	Nurse study coordinator
Cabello González, Carolina	Complejo Hospitalario de Navarra, Pamplona, Spain	Site investigator	Scheduling of evaluations
Cabo López, Iria	Complejo Hospitalario Universitario de Pontevedra (CHOP), Pontevedra, Spain	Site investigator /PI	Coordination at the center Evaluation of participants and/or data management
Caballol, Nuria	Consorci Sanitari Integral, Hospital Moisés Broggi, Sant Joan Despí, Barcelona, Spain.	Site investigator /PI	Coordination at the center Evaluation of participants and/or data management
Cámara Lorenzo, Ana	Hospital Clínic de Barcelona, Barcelona, Spain	Site investigator	Nurse study coordinator
Carrillo, Fátima	Hospital Universitario Virgen del Rocío, Sevilla, Spain	Site investigator	Evaluation of participants and/or data management
Carrillo Padilla, Francisco José	Hospital Universitario de Canarias, San Cristóbal de la Laguna, Santa Cruz de Tenerife, Spain	Site investigator /PI	Coordination at the center
Casas, Elena	Complejo Asistencial Universitario de Burgos, Burgos, Spain	Site investigator	Evaluation of participants and/or data management
Catalán, Maria Joé	Hospital Universitario Clínico San Carlos, Madrid, Spain	Site investigator /PI	Coordination at the center Evaluation of participants and/or data management
Clavero, Pedro	Complejo Hospitalario de Navarra, Pamplona, Spain	Site investigator	Evaluation of participants and/or data management
Cortina Fernández, A	Complejo Hospitalario Universitario de Ferrol (CHUF), Ferrol, A Coruña, Spain	Site investigator	Coordination of blood extractions
Cosgaya, Marina	Hospital Clínic de Barcelona, Barcelona, Spain	Site investigator	Evaluation of participants and/or data management
Cots Foraster, Anna	Institut d'Assistència Sanitària (IAS) - Instituí Cátala de la Salud. Girona, Spain	Site investigator	Evaluation of participants and/or data management
Crespo Cuevas, Ane	Hospital del Mar, Barcelona, Spain.	Site investigator	Evaluation of participants and/or data management
Cubo, Esther	Complejo Asistencial Universitario de Burgos, Burgos, Spain	Site investigator /PI	Coordination at the center Evaluation of participants and/or data management
De Deus Fonticoba, Teresa	Complejo Hospitalario Universitario de Ferrol (CHUF), Ferrol, A Coruña, Spain	Site investigator	Nurse study coordinator Evaluation of participants and/or data management
De Fábregues, Oriol	Hospital Universitario Vall d´Hebron, Barcelona, Spain	Site investigator /PI	Coordination at the center Evaluation of participants and/or data management
Díez Fairen, M	Hospital Universitari Mutua de Terrassa, Terrassa, Barcelona, Spain	Site investigator	Evaluation of participants and/or data management
Erro, Elena	Complejo Hospitalario de Navarra, Pamplona, Spain	Site investigator	Evaluation of participants and/or data management
Escalante, Sonia	Hospital de Tortosa Verge de la Cinta (HTVC), Tortosa, Tarragona, Spain	Site investigator /PI	Coordination at the center Evaluation of participants and/or data management
stelrich Peyret, Elena	Institut d'Assistència Sanitària (IAS) - Instituí Cátala de la Salud. Girona, Spain	Site investigator	Evaluation of participants and/or data management
Fernández Guillán, Noelia	Complejo Hospitalario Universitario de Ferrol (CHUF), Ferrol, A Coruña, Spain	Site investigator	Neuroimaging studies
Gámez, Pedro	Complejo Asistencial Universitario de Burgos, Burgos, Spain	Site investigator	Evaluation of participants and/or data management
Gallego, Mercedes	Hospital La Princesa, Madrid, Spain	Site investigator	Evaluation of participants and/or data management
García Caldentey, Juan	Centro Neurológico Oms 42, Palma de Mallorca, Spain	Site investigator /PI	Coordination at the center Evaluation of participants and/or data management
García Campos, Cristina	Hospital Universitario Virgen Macarena, Sevilla, Spain	Site investigator	Evaluation of participants and/or data management
García Moreno, Jose Manuel	Hospital Universitario Virgen Macarena, Sevilla, Spain	Site investigator /PI	Coordination at the center Evaluation of participants and/or data management
Gastón, Itziar	Complejo Hospitalario de Navarra, Pamplona, Spain	Site investigator /PI	Coordination at the center Evaluation of participants and/or data management
Guillén Fopiani, Desiré	Complejo Hospitalario Universitario de Pontevedra (CHOP), Pontevedra, Spain	Site investigator	Neuropsychologist; evaluation of participants
Gómez Garre, María del Pilar	Hospital Universitario Virgen del Rocío, Sevilla, Spain	Site investigator	Genetic studies coordination
Gómez Mayordomo, Víctor	Hospital Clínico San Carlos, Madrid, Spain	Site investigator	Evaluation of participants and/or data management
González Aloy, Javier	Institut d'Assistència Sanitària (IAS) - Instituí Cátala de la Salud. Girona, Spain	Site investigator	Evaluation of participants and/or data management
González Aramburu, Isabel	Hospital Universitario Marqués de Valdecilla, Santander, Spain	Site investigator	Evaluation of participants and/or data management
González Ardura, Jessica	Hospital Universitario Lucus Augusti (HULA), Lugo, Spain	Site investigator /PI	Coordination at the center Evaluation of participants and/or data management
González García, Beatriz	Hospital La Princesa, Madrid, Spain	Site investigator	Nurse study coordinator
González Palmás, Maria Josefa	Complejo Hospitalario Universitario de Pontevedra (CHOP), Pontevedra, Spain	Site investigator	Evaluation of participants and/or data management
González Toledo, Gabriel Ricardo	Hospital Universitario de Canarias, San Cristóbal de la Laguna, Santa Cruz de Tenerife, Spain	Site investigator	Evaluation of participants and/or data management
Golpe Díaz, Ana	Complejo Hospitalario Universitario de Ferrol (CHUF), Ferrol, A Coruña, Spain	Site investigator	Laboratory analysis coordination
Grau Solá, Mireia	Consorci Sanitari Integral, Hospital Moisés Broggi, Sant Joan Despí, Barcelona, Spain	Site investigator	Evaluation of participants and/or data management
Guardia, Gemma	Hospital Universitari Mutua de Terrassa, Terrassa, Barcelona, Spain	Site investigator	Evaluation of participants and/or data management
Hernández Vara, Jorge	Hospital Universitario Vall d´Hebron, Barcelona, Spain	Site investigator /PI	Coordination at the center Evaluation of participants and/or data management
Horta Barba, Andrea	Hospital de Sant Pau, Barcelona, Spain	Site investigator	Neuropsychologist; evaluation of participants
Idoate Calderón, Daniel	Complejo Hospitalario Universitario de Pontevedra (CHOP), Pontevedra, Spain	Site investigaor	Neuropsychologist; evaluation of participants
Infante, Jon	Hospital Universitario Marqués de Valdecilla, Santander, Spain	Site investigator /PI	Coordination at the center Evaluation of participants and/or data management
Jesús, Silvia	Hospital Universitario Virgen del Rocío, Sevilla, Spain	Site investigator	Evaluation of participants and/or data management
Kulievsky, Jaime	Hospital de Sant Pau, Barcelona, Spain	Site investigator /PI	Coordination at the center Evaluation of participants and/or data management
Kurtis, Mónica	Hospital Ruber Internacional, Madrid, Spain	Site investigator /PI	Coordination at the center Evaluation of participants and/or data management
Labandeira, Carmen	Hospital Álvaro Cunqueiro, Complejo Hospitalario Universitario de Vigo (CHUVI), Vigo, Spain	Site investigator	Evaluation of participants and/or data management
Labrador Espinosa, Miguel Ángel	Hospital Universitario Virgen del Rocío, Sevilla, Spain	Site investigator	Neuroimaging data analysis
Lacruz, Francisco	Complejo Hospitalario de Navarra, Pamplona, Spain	Site investigator	Evaluation of participants and/or data management
Lage Castro, Melva	Complejo Hospitalario Universitario de Pontevedra (CHOP), Pontevedra, Spain	Site investigator	Evaluation of participants and/or data management
Legarda, Inés	Hospital Universitario Son Espases, Palma de Mallorca, Spain	Site investigator /PI	Coordination at the center Evaluation of participants and/or data management
López Ariztegui, Nuria	Complejo Hospitalario de Toledo, Toledo, Spain	Site investigator /PI	Evaluation of participants and/or data management
López Díaz, Luis Manuel	Hospital Da Costa de Burela, Lugo, Spain	Site investigator	Evaluation of participants and/or data management
López Manzanares, Lydia	Hospital La Princesa, Madrid, Spain	Site investigator /PI	Coordination at the center Evaluation of participants and/or data management
López Seoane, Balbino	Complejo Hospitalario Universitario de Ferrol (CHUF), Ferrol, A Coruña, Spain	Site investigator	Neuroimaging studies
Lucas del Pozo, Sara	Hospital Universitario Vall d´Hebron, Barcelona, Spain	Site investigator	Evaluation of participants and/or data management
Macías, Yolanda	Fundación Hospital de Alcorcón, Madrid, Spain	Site investigator	Evaluation of participants and/or data management
Mata, Marina	Hospital Infanta Sofía, Madrid, Spain	Site investigator	Evaluation of participants and/or data management
Martí Andres, Gloria	Hospital Universitario Vall d´Hebron, Barcelona, Spain	Site investigator	Evaluation of participants and/or data management
Martí, Maria José	Hospital Clínic de Barcelona, Barcelona, Spain	Site investigator /PI	Coordination at the center Evaluation of participants and/or data management
Martínez Castrillo, Juan Carlos	Hospital Universitario Ramón y Cajal, Madrid, Spain	Site investigator /PI	Coordination at the center Evaluation of participants and/or data management
Martinez-Martin, Pablo	Centro Nacional de Epidemiología y CIBERNED, Instituto de Salud Carlos III. Madrid	Collaborator in statistical and methods analysis	Methods and statistical reviewer
McAfee, Darrian	University of Pennsylvania, Philadelphia	Collaborator in english style	English style reviewer
Meitín, Maria Teresa	Hospital Da Costa de Burela, Lugo, Spain	Site investigator	Evaluation of participants and/or data management
Menéndez González, Manuel	Hospital Universitario Central de Asturias, Oviedo, Spain	Site investigator /PI	Coordination at the center Evaluation of participants and/or data management
Méndez del Barrio, Carlota	Hospital Universitario Virgen del Rocío, Sevilla, Spain	Site investigator	Evaluation of participants and/or data management
Mir, Pablo	Hospital Universitario Virgen del Rocío, Sevilla, Spain	Site investigator /PI	Coordination at the center Evaluation of participants and/or data management
Miranda Santiago, Javier	Complejo Asistencial Universitario de Burgos, Burgos, Spain	Site investigator	Evaluation of participants and/or data management
Morales Casado, Maria Isabel	Complejo Hospitalario de Toledo, Toledo, Spain.	Site investigator	Evaluation of participants and/or data management
Moreno Diéguez, Antonio	Complejo Hospitalario Universitario de Ferrol (CHUF), Ferrol, A Coruña, Spain	Site investigator	Neuroimaging studies
Nogueira, Víctor	Hospital Da Costa de Burela, Lugo, Spain	Site investigator /PI	Coordination at the center Evaluation of participants and/or data management
Novo Amado, Alba	Complejo Hospitalario Universitario de Ferrol (CHUF), Ferrol, A Coruña, Spain	Site investigator	Neuroimaging studies
Novo Ponte, Sabela	Hospital Universitario Puerta de Hierro, Madrid, Spain.	Site investigator	Evaluation of participants and/or data management
Ordás, Carlos	Hospital Rey Juan Carlos, Madrid, Spain, Madrid, Spain.	Site investigator	Evaluation of participants and/or data management
Pagonabarraga, Javier	Hospital de Sant Pau, Barcelona, Spain	Site investigator	Evaluation of participants and/or data management
Isabel Pareés	Hospital Ruber Internacional, Madrid, Spain	Site investigator	Evaluation of participants and/or data management
Pascual-Sedano, Berta	Hospital de Sant Pau, Barcelona, Spain	Site investigator	Evaluation of participants and/or data management
Pastor, Pau	Hospital Universitari Mutade Terrassa, Terrassa, Barcelona, Spain	Site investigator	Evaluation of participants and/or data management
Pérez Fuertes, Aída	Complejo Hospitalario Universitario de Ferrol (CHUF), Ferrol, A Coruña, Spain	Site investigator	Blood analysis
Pérez Noguera, Rafael	Hospital Universitario Virgen Macarena, Sevilla, Spain	Site investigator	Evaluation of participants and/or data management
Planas-Ballvé, Ana	Consorci Sanitari Integral, Hospital Moisés Broggi, Sant Joan Despí, Barcelona, Spain	Site investigator	Evaluation of participants and/or data management
Planellas, Lluís	Hospital Clínic de Barcelona, Barcelona, Spain	Site investigator	Evaluation of participants and/or data management
Prats, Marian Ángeles	Institut d'Assistència Sanitària (IAS) - Instituí Cátala de la Salud. Girona, Spain	Site investigator	Evaluation of participants and/or data management
Prieto Jurczynska, Cristina	Hospital Rey Juan Carlos, Madrid, Spain, Madrid, Spain	Site investigator /PI	Coordination at the center Evaluation of participants and/or data management
Puente, Víctor	Hospital del Mar, Barcelona, Spain	Site investigator /PI	Coordination at the center Evaluation of participants and/or data management
Pueyo Morlans, Mercedes	Hospital Universitario de Canarias, San Cristóbal de la Laguna, Santa Cruz de Tenerife, Spain	Site investigator	Evaluation of participants and/or data management
Redondo, Nuria	Hospital La Princesa, Madrid, Spain	Site Investigator	Evaluation of participants and/or data management
Rodríguez Méndez, Luisa	Complejo Hospitalario Universitario de Ferrol (CHUF), Ferrol, A Coruña, Spain	Site investigator	Blood analysis
Rodríguez Pérez, Amparo Belén	Hospital General Universitario de Elche, Elche, Spain	Site investigator	Evaluation of participants and/or data management
Roldán, Florinda	Hospital Universitario Virgen del Rocío, Sevilla, Spain	Site investigator	Neuroimaging studies
Ruíz de Arcos, María	Hospital Universitario Virgen Macarena, Sevilla, Spain.	Site investigator	Evaluation of participants and/or data management
Ruíz Martínez, Javier	Hospital Universitario Donostia, San Sebastián, Spain	Site investigator	Evaluation of participants and/or data management
Sánchez Alonso, Pilar	Hospital Universitario Puerta de Hierro, Madrid, Spain	Site investigator	Evaluation of participants and/or data management
Sánchez-Carpintero, Macarena	Complejo Hospitalario Universitario de Ferrol (CHUF), Ferrol, A Coruña, Spain	Site investigator	Neuroimaging studies
Sánchez Díez, Gema	Hospital Universitario Ramón y Cajal, Madrid, Spain	Site investigator	Evaluation of participants and/or data management
Sánchez Rodríguez, Antonio	Hospital Universitario Marqués de Valdecilla, Santander, Spain	Site investigator	Evaluation of participants and/or data management
Santacruz, Pilar	Hospital Clínic de Barcelona, Barcelona, Spain	Site investigator	Evaluation of participants and/or data management
Santos García, Diego	CHUAC, Complejo Hospitalario Universitario de A Coruña	Coordinator of the Project	Coordination of the COPPADIS-2015
Segundo Rodríguez, José Clemente	Complejo Hospitalario de Toledo, Toledo, Spain	Site investigator	Evaluation of participants and/or data management
Seijo, Manuel	Complejo Hospitalario Universitario de Pontevedra (CHOP), Pontevedra, Spain	Site investigator /PI	Coordination at the center Evaluation of participants and/or data management
Sierra, María	Hospital Universitario Marqués de Valdecilla, Santander, Spain	Site investigator	Evaluation of participants and/or data management
Solano, Berta	Institut d'Assistència Sanitària (IAS) - Instituí Cátala de la Salud. Girona, Spain	Site investigator /PI	Coordination at the center Evaluation of participants and/or data management
Suárez Castro, Ester	Complejo Hospitalario Universitario de Ferrol (CHUF), Ferrol, A Coruña, Spain	Site investigator	Evaluation of participants and/or data management
Tartari, Juan Pablo	Hospital Universitari Mutua de Terrassa, Terrassa, Barcelona, Spain	Site investigator	Evaluation of participants and/or data management
Valero, Caridad	Hospital Arnau de Vilanova, Valencia, Spain	Site investigator	Evaluation of participants and/or data management
Vargas, Laura	Hospital Universitario Virgen del Rocío, Sevilla, Spain	Site investigator	Evaluation of participants and/or data management
Vela, Lydia	Fundación Hospital de Alcorcón, Madrid, Spain	Site investigator /PI	Coordination at the center Evaluation of participants and/or data management
Villanueva, Clara	Hospital Universitario Clínico San Carlos, Madrid, Spain	Site investigator	Evaluation of participants and/or data management
Vives, Bárbara	Hospital Universitario Son Espases, Palma de Mallorca, Spain	Site investigator	Evaluation of participants and/or data management
Villar, Maria Dolores	Hospital Universitario de Canarias, San Cristóbal de la Laguna, Santa Cruz de Tenerife, Spain	Site investigator	Evaluation of participants and/or data management

## Data Availability

The protocol and the statistical analysis plan are available on request. Deidentified participants data are not available for legal and ethical reasons.

## Appendix

### A. COPPADIS Study Group

The authors in the COPPADIS Study Group have been listed in Table 5.

## Abbreviations

ADLS: Schwab and England Activities of Daily Living Scale
BDI: Beck depression inventory-II
EUROHIS-QOL8: European Health Interview Survey-Quality of Life 8 Item-Index
FOGQ: Freezing of gait questionnaire
H&Y: Hoenh and Yahr
NMS: Nonmotor symptoms
NMSB: Nonmotor symptoms burden
NMSS: Nonmotor symptoms scale
NPI: Neuropsychiatric inventory
PD: Parkinson's disease
PD-CRS: Parkinson's Disease Cognitive Rating Scale
PDQ-39SI: 39-Item Parkinson's Disease Quality of Life Questionnaire Summary Index
PDSS: Parkinson's Disease Sleep Scale
QoL: Quality of life
QUIP-RS: Questionnaire for Impulsive-Compulsive Disorders in Parkinson's Disease-Rating Scale
UPDRS: Unified Parkinson's Disease Rating Scale
VAFS: Visual Analog Fatigue Scale
VAS pain: Visual Analog Scale Pain.

## Supplementary Materials

Supplementary Materials Table 1 SM. Disease-related characteristics, motor and nonmotor symptoms, and autonomy for activities of daily living and quality of life in PD patients (*n*=603). Table 2 SM. Multiple regression model for PDQ-39SI as dependent variable.

## References

[1] Olanow C. W., Obeso J. A. (2012). The significance of defining preclinical or prodromal Parkinson’s disease. *Movement Disorders*.

[2] Hoehn M. M., Yahr M. D. (1967). Parkinsonism: onset, progression, and mortality. *Neurology*.

[3] Goetz C. G., Poewe W., Rascol O. (2004). Movement disorder society task force report on the hoehn and yahr staging scale: status and recommend the authorsions the movement disorder society task force on rating scales for Parkinson’s disease. *Movement Disorders*.

[4] Fanhn S., Elton R. L., Members of the UPDRS Development Committee, Fahn S., Marsden C. D., Calne D. B., Goldstein M. (1987). Unified Parkinson’s disease rating scale. *Recent developments in Parkinson’s disease*.

[5] Goetz C. G., Tilley B. C., Shaftman S. R. (2008). Movements disorder society-sponsored revision of the unisied partinson’s disease rating scale (mds-updrs): scale presentation and clinimetric testing results. *Movement Disorders*.

[6] Chaudhuri K. R., Martinez-Martin P., Schapira A. H. V. (2006). International multicenter pilot study of the first comprehensive self-completed nonmotor symptoms questionnaire for Parkinson’s disease: the NMSQuest studyInternational multicenter pilot study of the first. *Movement Disorders*.

[7] Chaudhuri K. R., Martinez-Martin P., Brown R. G. (2007). The metric properties of a novel non-motor symptoms scale for Parkinson´s disease: results from an international pilot studyThe metric properties of a novel non-motor symptoms scale for Parkinson’s disease: Results from an international pilot study. *Movement Disorders*.

[8] Santos-García D., de la Fuente-Fernández R. (2013). Impact of non-motor symptoms on health-related and perceived quality of life in Parkinson’s disease. *Journal of the Neurological Sciences*.

[9] Martinez-Martin P., Rodriguez-Blazquez C., Kurtis M. M., Chaudhuri K. R. (2011). The impact of non-motor symptoms on health-related quality of life of patients with Parkinson’s disease. *Movement Disorders*.

[10] Martinez-Martin P., Ray Chaudhuri K. (2018). prehensive grading of Parkinson’s disease using motor and non-motor assessments: addressing a key unmet need. *Expert Review of Neurotherapeutics*.

[11] Ray Chaudhuri K., Rojo J. M., Schapira A. H. (2013). A proposal for a comprehensive grading of Parkinson’s disease severity combining motor and non-motor assessments: meeting an unmet need. *PLoS One*.

[12] Santos-García D., Mir P., Cubo E. (2016). COPPADIS-2015 (Cohort of Patients with Parkinson’s disease in Spain, 2015), a global--clinical evaluations, serum biomarkers, genetic studies and neuroimaging--prospective, multicenter, non-interventional, long-term study on Parkinson’s disease progression. *BMC Neurology*.

[13] Santos García D., Jesús S., Aguilar M. (2019). COPPADIS-2015 (COhort of Patient’s with Parkinson’s disease in Spain, 2015): An ongoing global Parkinson’s d.isease Pr.oject about d.isease progression with more than 1,000 subjects included. results from the baseline evaluation. *European Journal of Neurology*.

[14] Jenkinson C., Fitzpatrick R., Peto V., Greenhall R., Hyman N. (1997). The Parkinson’s disease questionnaire (PDQ-3.9): development and validation of a Parkinson’s disease summary index score. *Age and Ageing*.

[15] Santos García D., de Deus Fonticoba T., Suárez Castro E. (2019). Non-motor symptoms burden, mood, and gait problems are the most significant factors contributing to a poor quality of life in non-demented Parkin.son’s dis.ease patien.ts: Results from the COPPADIS Study Cohort. *Parkinsonism & Related Disorders*.

[16] Rocha N. S. d., Power M. J., Bushnell D. M., Fleck M. P. (2012). The EUROHIS-QOL 8-item index: comparative psychometric properties to its parent WHOQOL-BREF. *Value in Health*.

[17] Schwab R. S., England A. C. (1969). *Parkinson’s disease*.

[18] Santos-García D., de Deus-Fonticoba T., Suárez Castro E. (2020). The impact of freezing of. gait on function.al dependency in Parkinson’s disease with regard to motor phenotype. *Neurological Sciences*.

[19] Burn D. J., Landau S., Hindle J. V., Samuel M., Wilson K. C., Hurt C. S. (2012). Parkinson’s disease motor subtypes and mood. *Movement Disorders*.

[20] Todorova A., Jenner P., Ray Chaudhuri K. (2014). Non-motor Parkinson’s: integral to motor Parkinson’s, yet often neglected. *Practical Neurology*.

[21] Sauerbier A., Jenner P., Todorova A. (2016). Non motor subtypes and Parkinson’s disease. *Parkinsonism & Related Disorders*.

[22] Prakash K. M., Nadkarni N. V., Lye W.-K., Yong M.-H., Tan E.-K. (2016). The impact of non-motor symptoms on the quality of life of Parkinson’s disease patients: a longitudinal study. *European Journal of Neurology*.

[23] Arotcarena M. L., Dovero S., Prigent A. (2020). Bidirectional gut-to-brain and brain-to-gut propagation of synucleinopathy in non-human primates. *Brain*.

[24] Horsager J., Andersen K. B., Knudsen K. (2020). Brain-first versus body-first Parkinson’s disease: a multimodal imaging case-control study. *Brain*.

[25] Antonini A., Bauer L., Dohin E. (2015). Effects of rotigotine transdermal patch in patients with Parkinson’s disease presenting with non-motor symptoms - results of a double-blind, randomized, placebo-controlled trial. *European Journal of Neurology*.

[26] Seppi K., Ray Chaudhuri K., Coelho M. (2019). Update on treatments for nonmotor symptoms of Parkinson’s disease-an evidence‐based medicine review. *Movement Disorders*.

[27] Peball M., Krismer F., Knaus H. G. (2020). Non‐Motor Symptoms in Parkinson’s Disease are Reduced by Nabilone. *Annals of Neurology*.

[28] Armañanzas R., Bielza C., Chaudhuri K. R., Martinez-Martin P., Larrañaga P. (2013). Unveiling relevant non- Parkinson’s disease severity symptoms using a machine learning approach. *Artificial Intelligence in Medicine*.

[29] Chaudhuri K. R., Schrag A., Weintraub D., Rizos A. (2020). The movement disorder society nonmotor rating scale: Initial validation study. *Movement Disorders*.

[30] Martínez-Fernández R., Schmitt E., Martinez-Martin P., Krack P. (2016). The hidden sister of motor flucsuations in Parkinson’s disease: A review on nonmotor fluctuations. *Movement Disorders*.

[31] Expósito-Ruiz I., Suárez-Castro E., Santos-García D. Relación entre calidad de vida, Hoehn&Yahr y síntomas no motores: el porqué de usar una escala que combine el estadio motor y la afectación no motora en pacientes con enfermedad de Parkinson. Oral Communication.

