# Supplementary material for: Staging Parkinson's Disease Combining Motor and Nonmotor Symptoms Correlates with Disability and Quality of Life
Source: Parkinsons Dis. 2021 May 13;2021:8871549. doi: 10.1155/2021/8871549 (PMC8140853; doi:10.1155/2021/8871549)
Supplement: Supplementary Materials — Table 1 SM. Disease-related characteristics, motor and nonmotor symptoms, and autonomy for activities of daily living and quality of life in PD patients (n=603). Table 2 SM. Multiple regression model for PDQ-39SI as dependent variable. [file 8871549.f1.zip › 8871549.f1/Table.1SM.H&Y.NMSB.docx]

**Table 1. SM.** Disease related characteristics, motor and non-motor symptoms, autonomy for activities of daily living and quality of life in PD patients (n=603).

| Age  Males (%)  Disease duration (years)  L-dopa eq. daily dose (mg)  Motor phenotype (%)  - Tremoric dominant  - PIGD  - Indeterminate  Hoehn & Yahr  - Stage 1 (%)  - Stage 2 (%)  - Stage 3 (%)  - Stages 4 – 5 (%)  UPDRS-III  UPDRS-IV  Motor fluctuations (%)  Dyskinesia (%)  FOG-Q  - Patients with falls (%  PD-CRS  - Cognitive impairment (PD-CRS ≤ 84) (%)  NMSS  - Mild NMS burden (NMSS 1-20) (%)  - Moderate severe burden (NMSS 21-40) (%)  - Severe NMS burden (NMSS 41-70) (%)  - Very severe NMS burden (NMSS > 70) (%)  BDI-II  - Depressive symptoms (%)  - Major depression (%)  - Minor depression (%)  - Subthreshold depression (%)  NPI  QUIP-RS  PDSS  VAS-PAIN  - Patients with pain (%)  VASF − physical  VASF – mental  ADLS  - Patients with functional dependency (%)  PDQ-39SI  PQ-10  EUROHIS-QOL8 | 62.7 ± 8.9  59.5  5.7 ± 4.5  576.1 ± 421.6  44.4  40.6  15  1.9 ± 0.5  22.9  67.5  8.1  1.5  22.8 ± 11.2  2.1 ± 2.5  37.1  19.7   3.9 ± 4.6  13.8  90.8 ± 16  32.1  46.7 ± 38.2  26.9  28.8  23.2  21.1  8.9 ± 7.4  51.7  16.7  17.1  18.2  6.4 ± 8.3  4.5 ± 8.3  115 ± 26.3  2.7 ± 2.9  56.9  3.1 ± 2.8  2.2 ± 2.6  88 ± 10.5  10.1  17.6 ± 13.7  7.2 ± 1.6  3.8 ± 0.6 |
| --- | --- |

The results represent percentages or mean ± SD. Data about H&Y and UPDRS-III are during the OFF state (first thing in the morning without taking medication in the previous 12 hours).

ADLS, Schwab and England Activities of daily living Scale); BDI, Beck Depression Inventory-II; NMSS, Non-Motor Symptoms Scale; NPI, Neuropsychiatric Inventory; PD, Parkinson´s disease; PD-CRS, Parkinson’s Disease Cognitive Rating Scale; PDSS, Parkinson’s Disease Sleep Scale; PIGD, Postural Inestability Gait Dificulty; QUIP-RS, Questionnaire for Impulsive-Compulsive Disorders in Parkinson’s Disease-Rating Scale; UPDRS, Unified Parkinson’s Disease Rating Scale; VAFS, Visual Analog Fatigue Scale; VAS-Pain, Visual Analog Scale-Pain.
