# Supplementary material for: Staging Parkinson's Disease Combining Motor and Nonmotor Symptoms Correlates with Disability and Quality of Life
Source: Parkinsons Dis. 2021 May 13;2021:8871549. doi: 10.1155/2021/8871549 (PMC8140853; doi:10.1155/2021/8871549)
Supplement: Supplementary Materials — Table 1 SM. Disease-related characteristics, motor and nonmotor symptoms, and autonomy for activities of daily living and quality of life in PD patients (n=603). Table 2 SM. Multiple regression model for PDQ-39SI as dependent variable. [file 8871549.f1.zip › 8871549.f1/Table.2.SM..doc]

Table 2.SM. Multiple regression model for PDQ-39SI as dependent variable.

|  | Adjusted  R-squared | Β standardized coefficient | CI 95% | *p* value |
| --- | --- | --- | --- | --- |
| Age  Gender  Disease duration  levodopa equivalent daily dose  UPDRS-IV  FOGQ  BDI-II  HY.NMSB | 0.591 | -0.053  0.0.51  -0.015  0.024  0.109  0.328  0.474  0.089 | -0.165, 0.103  -0.104, 2.958  0.63, 0.97  -0.238, 0.147  0.211, 1.002  0.764. 1.090  0.763, 0.983  0.098, 0.770 | 0.060  0.068  0.645  0.389  **0.003**  **<0.0001**  **<0.0001**  **0.011** |

BDI-II, Beck Depression Inventory-II; FOGQ, Freezing Of Gait Questionnaire; H&Y, Hoehn & Yahr scale; NMSB, Non-Motor Symptoms Burden; UPDRS, Unified Parkinson’s Disease Rating Scale.
